# URS DataBase: universe of RNA structures and their motifs

**DOI:** 10.1093/database/baw085

**Published:** 2016-05-30

**Authors:** Eugene Baulin, Victor Yacovlev, Denis Khachko, Sergei Spirin, Mikhail Roytberg

**Affiliations:** ^1^Laboratory of Applied Mathematics, Institute of Mathematical Problems of Biology, Russian Academy of Sciences, Pushchino, Moscow Region 142290, Russia; ^2^Department of Algorithms and Technology of Programming, Faculty of Innovations and High Technology, Moscow Institute of Physics and Technology (State University), Dolgoprudny, Moscow Region 141700, Russia; ^3^Department of Big Data and Information Retrieval, Faculty of Computer Science, National Research University Higher School of Economics, Moscow 101000, Russia; ^4^Department of Mathematical Methods in Biology, Belozersky Institute of Physico-Chemical Biology, Lomonosov Moscow State University, Moscow 119992, Russia

## Abstract

The Universe of RNA Structures DataBase (URSDB) stores information obtained from all RNA-containing PDB entries (2935 entries in October 2015). The content of the database is updated regularly. The database consists of 51 tables containing indexed data on various elements of the RNA structures. The database provides a web interface allowing user to select a subset of structures with desired features and to obtain various statistical data for a selected subset of structures or for all structures. In particular, one can easily obtain statistics on geometric parameters of base pairs, on structural motifs (stems, loops, etc.) or on different types of pseudoknots. The user can also view and get information on an individual structure or its selected parts, e.g. RNA–protein hydrogen bonds. URSDB employs a new original definition of loops in RNA structures. That definition fits both pseudoknot-free and pseudoknotted secondary structures and coincides with the classical definition in case of pseudoknot-free structures. To our knowledge, URSDB is the first database supporting searches based on topological classification of pseudoknots and on extended loop classification.

**Database URL**: http://server3.lpm.org.ru/urs/

## Introduction

RNA seems to be the least investigated class of irregular biopolymers. The role of the messenger, transfer and ribosomal RNA is well known, but the role of various non-coding RNA in the regulation of intracellular processes, including the regulation of gene expression, requires further study, see the review (1). RNA function is closely connected with its spatial structure. Until recently RNA structures were mostly studied from the perspective of pseudoknot-free RNA. The programs predicting pseudoknot-free secondary structure of RNA, see (2–6) and review (7), rely on the Nearest Neighbor Model (8, 9). The tools predicting pseudoknotted secondary structures or three-dimensional (tertiary) structures of RNA (10–14) have significantly lower quality.

For classical secondary structures there is a common classification of structural elements (15, 16), however, there is no such classification for arbitrary secondary structures.

Currently, there are several databases that contain information on RNA structures and structural motifs. RNA Frabase 2.0 (17) stores RNA secondary structures and their structural elements (base pairs, stems, loops). Its search engine allows queries containing RNA sequence and secondary structure patterns and a wide range of parameters of structural elements. RNA 3D Motif Atlas (18) provides detailed information on 3D RNA motifs and their components (base pairs, base stacking, base–phosphate interactions). All 3D motifs are clustered and the data are manually curated. Nucleic Acid Database (19) provides a search engine using a wide set of parameters (see Case 2 in the Discussion). RNA Strand (20) stores RNA secondary structures including those with unknown 3D structure. It allows the user to search using various features of secondary structure itself and secondary structure elements including pseudoknots. Output of the requested statistics on the selected structures is also available. RNA Bricks (21) stores RNA 3D motifs and provides information about local environments of the collected motifs, including contacts with proteins and metal ions. It also stores data on contacts between symmetry mates in crystals and between molecules from split PDB entries. RNA CoSSMos (22) stores secondary structure motifs such as mismatches, internal loops, hairpins and bulges and provides systematic search for these motifs. PseudoBase ++ (23) stores pseudoknots and provides their detailed descriptions. A search engine is also available. NPIDB (24) and PRIDB (25) store RNA–protein interactions along with annotations of protein secondary structure.

Despite the availability of a variety of databases dedicated to RNA structures at the moment, to our knowledge, there is no database that contains all available 3D structures of RNA and at the same time contains annotations of their main structural elements (stems, loops, pseudoknots, etc.) and allows an annotation-based search. The vast majority of existing databases are limited to pseudoknot-free RNA structures.

The presented database URSDB aims to fill the above gaps. Its main features are (i) detailed annotation of pseudoknot-related structural elements that naturally generalizes the annotation used for pseudoknot-free structures along with possibility to perform search using this annotation and (ii) a user-friendly web interface allowing, in particular, to select subsets of structures to be further analyzed.

Our database is a set of thoroughly verified and uniformly annotated structures. Such dataset is necessary to develop and evaluate tools dealing with RNA structures, e.g. predicting RNA secondary structures, determining RNA-binding regions in proteins, comparing related RNA molecules, etc. For example, the database can help to create knowledge-based potentials for various classes of RNA–RNA or RNA–protein interactions. Another example is usage of the URS database within testing software for secondary structure prediction. A prediction program can fail on RNA having certain types of secondary structure. To reveal that, a developer needs training sets with different characteristics. At the same time a scientist studying RNA can use the database to extract the structures with desired features, e.g. containing pseudoknots with given signatures, and obtain the characteristics of the structures.

## Materials and methods

### Input data

RNA-containing structures were extracted from the PDB in mmCIF format; each file was divided into models. The base pairs (both canonical and non-canonical) and dinucleotide steps were annotated using the DSSR program from 3DNA toolkit (26). We also exploited detailed information provided by DSSR on given elements such as geometric parameters, types according to different classifications and various details on base conformations.

### Implementation

To create the database we designed a special program package; the package was implemented in Python 3 and consists of 28 independent modules. The package output is a set of text files in MySQL format. The database is powered by MySQL server 5.1. The database web interface is developed as a collection of Python 2.7 CGI scripts along with HTML pages and JavaScript code. Windows of individual structures and motifs use Sencha Ext JS framework (http://www.sencha.com/products/extjs/). Interactive 3D representations of structures and motifs are displayed using JSmol (http://sourceforge.net/projects/jsmol/).

### Terminology

The database relies on refined definitions of stems, loops, pseudoknots and their parts. For exact definitions see http://server3.lpm.org.ru/urs/struct.py?where=3&#def. Below we give some examples and brief explanations.

### Stems and ECRs

A Stem is a sequence of base pairs of the form (*i*, *j*), (*i* + 1, *j*−1), …, (*i* + *k*, *j*−*k*) where *k* > 0 and each number denotes a nucleotide in the corresponding position of a chain and two nucleotides in a pair are connected with hydrogen bonds. We consider several types of stems. In case of *standard* stem all base pairs are supposed to be Watson–Crick pairs or Wobble (GU) pairs.

All definitions below (wings, loops, etc.) are related to standard stems. However, they can be applied to stems of arbitrary type. Hereinafter ‘base pairs’ means complementary (i.e. Watson-Crick or Wobble G-U) base pairs.

**Figure 1.**
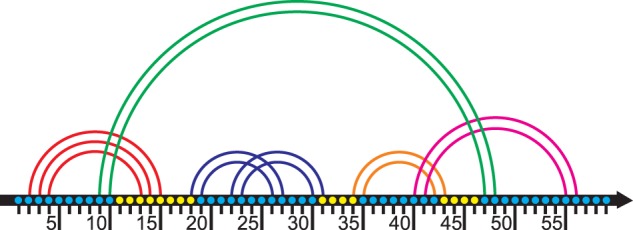
The stem H and its loop. Each base is represented with a dot; bases are enumerated from 1 to 57. Stems are represented with arcs. The stem H (in green) has the left wing at positions 9–10 and the right wing at positions 47–48. There are two H-ECR’s (see ‘Loops’ subsection below) inside H, the pseudoknotted nested ECR at positions 18–31, see blue arcs, and the ECR at positions 34–43, see the orange arc. The loop related to the stem H comprises positions 11–46 except at position lying inside H-related ECRs. Thus the loop of the stem H has the following structure: a side 11–17; a face of the pseudoknotted ECR {18, 31}, a side 32–33; a face of the stem {34; 43}, a side 44–46. Note that the side 11–17 contains a wing 13–15. Therefore the loop is a pseudoknotted multiple junction. The figure is prepared based on one of the figures from the website www.e-rna.org.

The first pair (*i*, *j*) in the stem (*i*, *j*), (*i* + 1, *j* − 1),…, (*i* + *k*, *j* − *k*) is called the external pair or the face of the stem. The last pair (*i* + *k*, *j* − *k*) is called the internal pair of the stem. The fragment [*i*, *i* + *k*] of an RNA chain is called the left wing of the stem, and the fragment [*j* − *k*, *j*] is called the right wing. We say that the base pair (m, n) **crosses** a stem with the face (*i*, *j*) if *m* < *i* < *n* < *j* or *i* < *m* < *j* <* n*, see Figure 1.

**Figure 2.**
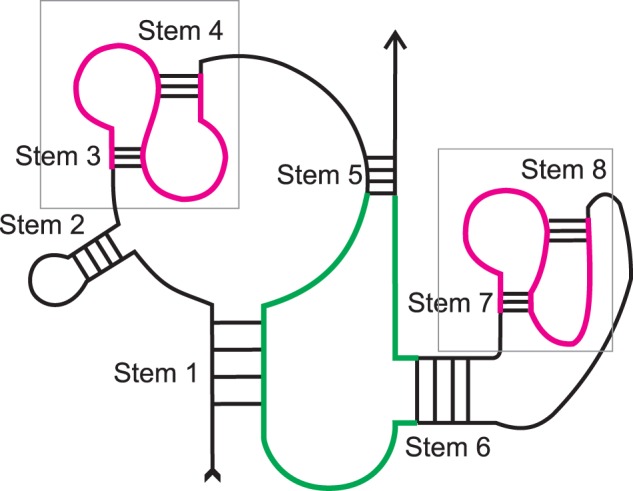
Loops of different stems can be of different types. Stem 1 – pseudoknotted multiple junction; Stem 2 – classical hairpin; Stems 3, 4, 7, 8 – pseudoknotted hairpins; Stem 5 – pseudoknotted internal loop; Stem 6 – isolated internal loop. Boxes and the structures outlined in purple highlight pseudoknots. The loop of stem 5 is shown in green.

**Figure 3.**
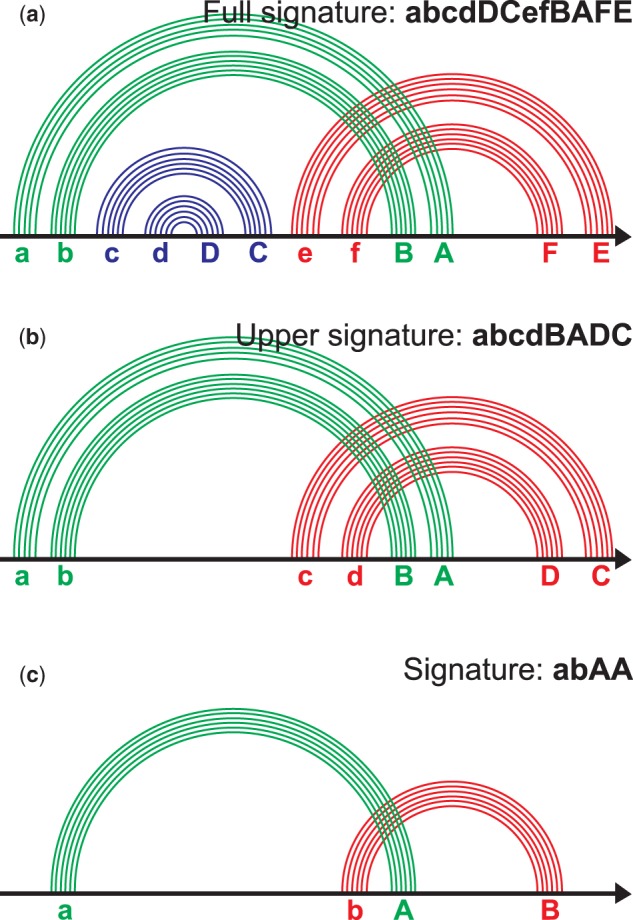
Signature of the pseudoknotted ECR. (**a**) The ECR contains six stems; each stem is labeled with a letter (see the text). The word abcdDCefBAFE composed of such letters is a full signature of the pseudoknot. (**b**) The nested stems named cC and dD at (a) are removed. The letters for the remaining stems are reassigned. The word abcdBADC is an upper signature of the pseudoknot. (**c**) We combine each family of parallel stems into one arc. The letters are reassigned. The word abAB is a signature of the pseudoknot. The figure is prepared after the site www.e-rna.org.

The next definition basically follows (27) but the terminology is slightly changed. Informally speaking, a closed region is an RNA fragment without bases paired to bases outside of the fragment. An Elementary Closed Region (ECR) is a closed region [i, j] that cannot be divided into smaller closed regions. See Figures 1–3. To be more formal, an Elementary Closed Region (ECR) is a segment [*i*, *j*] such that:

1. *i* < *j*
2. There is no base pairs (*k*, l) such that (*i* ≤ *k* ≤ *j*; *l* > *j*) or (*k* < *i*; *i* ≤ *l* ≤ *j*);
3. There is no t between i and j such that [*i*, *t*] or [*t*, *j*] satisfy the conditions 1) and 2);
4. Either the base pair (*i*, *j*) is the face of a stem or there are bases x and y inside the fragment [*i*, *j*] such that (*i*, *x*) and (*y*, *j*) are faces of two stems.

An ECR [*i*, *j*] is called a classical ECR if (*i*, *j*) is the face of a stem; otherwise the ECR is called a pseudoknotted ECR or a pseudoknot.

An ECR [k, l] is a nested ECR of an ECR [*i*, *j*] if *i* < *k* < *l* < *j* and there are no other ECR [*m*, *n*] such that *i* < *m* < *k* < *l* < *n* < *j*.

For more definitions see http://server3.lpm.org.ru/urs/struct.py?where=3&#def-basic2.

### Loops

To describe RNA secondary structure we use a definition of a loop that generalizes the definition used in Nearest Neighbor Model (9, 10). Our definition relies on the following generalization of ECR. Let H be a stem with the internal pair (*r*, *s*). An H-related Elementary Closed Region (H-ECR) is a segment [*i*, *j*] such that:

1. *r* < *i* < *j* < *s*;
2. There is no base pair (*k*, *l*) such that (*i* ≤ *k* ≤ *j*; *j*< *l* < *s*) or (*r* < *k* < *i*; *i* ≤ *l* ≤ *j*);
3. There is no t between i and j such that [*i*, *t*] or [*t*, *j*] satisfy the conditions 1) and 2);
4. Either the base pair (*i*, *j*) is the face of a stem or there are bases x and y inside the fragment [*i*, *j*] such that (*i*, *x*) and (*y*, *j*) are faces of two stems.

Let H be a stem and (*i*, *j*) its internal pair.

The position *t* is internal to stem H (synonym: lies inside H), if *i* < *t* < *j*. Fragment of chain is internal to stem H (synonym: lies inside H), if all its positions are internal to stem H. Stem H1 lies inside the stem H (is internal to H), if all the positions of its wings are internal to H.

The position *t* belongs to the stem H, if it is internal to H and there is no stem H1, lying inside H, such that *x* < *t* < *y*, where (*x*, *y*) is the external pair (face) of H1.

Loop of the stem H is the set of all positions that belong to stem H.

Our loops, as well as loops according to NNM, are in one to one correspondence with stems. Informally speaking the loop of a stem H with the internal pair (*r*, *s*) is the fragment [*r* + 1, *s* − 1] with excluded H-ECRs. For technical reasons we consider faces of excluded ECRs as parts of the loop. See Figures 1–3 and http://server3.lpm.org.ru/urs/struct.py?where=3&#def-stems-loops for further information.

If the structure is pseudoknot-free, each loop in terms of our definitions is a loop in terms of the Nearest Neighbor Model (NNM) and vice versa, but our definitions also can be applied to pseudoknots. In the latter case the loop may contain wings and, along with ends of stems (‘faces’), may contain faces of pseudoknotted fragments, see stems 1 and 6, Figure 2.

Our loops, as well as loops according to NNM, are in one to one correspondence with stems. The definition of pseudoknotted loop from the paper of B. Rastegari and A. Condon (27) does not follow this rule; therefore loops of Rastegari-Condon have more complicated structure and in general can be divided in several loops by our definition.

We divide a loop into sides separated by faces, see Figure 1.

In our terms the loops from NNM classification can be described as follows:

A loop is a hairpin if it does not contain faces and, therefore, has a single side. A loop is an internal loop if it contains exactly one face and therefore has two sides. A loop is a multiple junction if it contains more than one face and therefore more than two sides. An internal loop is a bulge if one of its sides is of zero length.

We also consider an additional classification of loops. A loop is a classical loop if it does not contain faces of pseudoknots and wings. A loop is called isolated if it does not contain wings and is called pseudoknotted otherwise (see Figure 2). A stem is pseudoknotted if its loop is pseudoknotted.

### Pseudoknot signatures

Definition of a pseudoknot or a pseudoknotted ECR was given in the Terminology section, see Figure 3. To classify pseudoknots we employ a two stage reduction process that includes (i) removing all nested ECRs and (ii) collapsing all base pairs of consecutive stems into one arc (Figure 3c); the notion of arc corresponds to a band from (27). Each arc is assigned with a letter, its left end is assigned with a small letter and the right end is assigned with the corresponding capital one, e.g. a and A, b and B, etc. The letters are assigned to arcs in alphabetical order from left to right according to position of an arc’s left end, see Figure 3. A *pseudoknot signature* of the ECR is a word composed of the letters assigned to the arcs’ ends; the order of the letters corresponds to order of positions of arcs’ ends. For example, the signature of an H-knot is abAB, signature of kissing hairpins is abAcBC and the signature of a triple knot is abcABC. For more details see http://server3.lpm.org.ru/urs/struct.py?where=3&#def-basic2.

The signature definition coincides with the definition from (28); description of pseudoknots via signatures in some form can be found in (29–32). To our knowledge the classification has not been implemented in RNA-related databases.

## Results

### Content of the database

The database contains 2935 RNA-containing structures from PDB, 7718 RNA chains (only one model per PDB entry was considered), 1 314 360 base pairs of different types and 5130 pseudoknots.

URSDB is a relational database powered by MySQL server. The database consists of 51 tables. The tables are divided into four groups: (1) tables of data stored in PDB (chains, residues, atoms, etc.), (2) tables of data from DSSR output (base-pairs, dinucleotide steps, helices, etc.), (3) tables of structural motifs compiled using our program package (threads, wings, loops, links, stems, pseudoknots, etc.), (4) auxiliary tables (parallel stems, RNA-protein H-bonds, etc.).

### Web interface

#### The database web interface allows users

1. to select a set of PDB entries;
2. to get numerical characteristics of structural elements for a selected subset of structures, for the entire database, for the non-redundant PDB list (16) or for an individual structure and
3. to analyze the structural elements of the chosen PDB entry or of the selected subset of entries.

The home page contains the main menu and a brief description of the database; the menu in particular contains links to two query pages, ‘Structures’ and ‘Statistics’ see below.

### Search for structures

The interface supports a wide range of elementary queries related to general information on a PDB entry, molecules, RNA patterns, base pairs, etc. An RNA pattern can be described with a sequence fragment, dot-bracket notation, pseudoknot signature or ECR pattern. The latter contains descriptions of all stems within the ECR. Furthermore, the user can also request the presence or absence of loops of various types, pseudoknots, etc. See http://server3.lpm.org.ru/urs/struct.py?where=3#set-rest-pat for details. In general, a user’s query can be an arbitrary disjunction (‘or’ junction) of conjunctions (‘and’ junctions) of elementary queries.

The user can edit previous queries, perform a search in previous search results or add new results to the results of a previous search. The search result is presented as a table; each line corresponds to a found structure. A user can specify structural information to be shown in a line and sort the table. By clicking an individual structure in the table, the user can activate a special window to analyze the structure, see below.

### Statistics

The ‘Statistics’ page allows users to view statistics related to structural elements, e.g. chains, base pairs, links, stems, loops, pseudoknots, multiplets and RNA–Protein hydrogen bonds. The request may be carried out in four modes, for the entire database, for the selected set of structures, for the non-redundant PDB list and for a selected PDB entry. When the request is processed and initial results are obtained one can continue using the ‘Filter’ field of the ‘Statistics’ page. The field allows setting various parameters of structural element to filter the results. Another possibility is to obtain the table containing the full list of structural elements meeting the given conditions. Clicking an element within the list one activates a window containing the detailed information on the element allowing viewing the element itself or the whole structure. For more information see the Help page at http://server3.lpm.org.ru/urs/struct.py?where=3&#stat.

### Analysis of an individual structure and its elements

Analysis of an individual PDB entry or its structural elements is performed in a special window. The window consists of two parts; the right part contains a JSmol viewer, the left part contains detailed information on the entry and its structural elements, e.g. chains, base pairs, stems, loops and pseudoknots. The window may show a whole structure or a structural element. The structure window can be activated using the result table of the ‘Structures’ page. The structural element window can be activated from the result table of the ‘Statistics’ page.

See http://server3.lpm.org.ru/urs/struct.py?where=3#using-example for the example of a typical URS session.

## Discussion

In this section, we will discuss possible applications of the database and its web interface. In our opinion the main advantage of URSDB is a possibility to work with various types of structural elements, e.g. stems, single-stranded fragments, all types of loops, base pairs, pseudoknots, etc. This allows one to easily collect different types of statistical data. From the other hand our web interface may serve as a convenient tool for search for RNA structures and its motifs and for construction of its subsets, and to pick up needed individual cases.

Below we consider two cases of using URSDB and its web interface named URS. The first example is related to a collection of statistics, and the second one demonstrates advantages of the URS interface.

### Case 1: statistics of tertiary base–base interactions

We used URSDB to collect data on tertiary interactions with respect to their belonging to the secondary structure motifs (stems or loops). We were inspired by work (33) where authors analyzed tertiary interactions (both local and long-range) from *Escherichia coli* 16S ribosomal RNA.

We analyzed RNA tertiary interactions separately in pseudoknots and in classical structures. As the dataset we used a non-redundant PDB list without resolution cutoff (16). Each interaction was marked by three different labels: (1) Type according to Leontis–Westhof classification (34); (2) Type according to secondary motifs which nucleotides belong to [Helix–Helix (HH), Loop–Helix (LH) or Loop–Loop (LL)]; and (3) is it Local (belongs to the same or adjacent secondary motifs) or Long-Range (otherwise).

As one can see from Table 1, there are some significant differences between pseudoknots and classical structures. Local interactions inside loops in classical structures constitute almost 82% of all interactions whereas it is only 65.5% in pseudoknots. Another significant difference is an increased amount of long-range interactions involving helices in pseudoknots (52.3 vs. 32.3%). Also one can observe differences in patterns of distribution of various types of long-range interactions in pseudoknots and in classical structures. As for local interactions, the patterns are very similar.

**Table 1.** Tertiary interactions in classical RNA structures and in pseudoknots

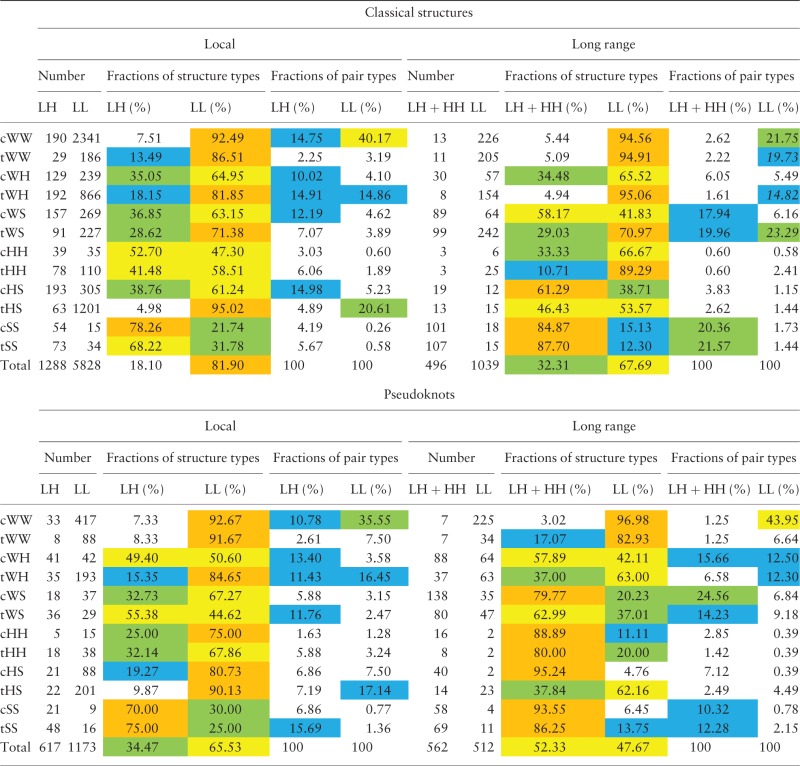

HL stands for Helix-Loop, HH stands for Helix-Helix, LL stands for Loop-Loop. Local interactions are the interactions inside one structural element or between two adjacent elements (e.g. between hairpin loop and its stem); other interactions are considered as long-range interactions. Each line corresponds to an interaction type. The cells contain corresponding (i) number of pairs of the type (‘Number of pairs’); (ii) fraction of LL and non-LL pairs among all pairs of the type (‘Fractions of structure types’); (iii) fraction of LL or non-LL pairs of the type among all LL or non-LL pairs (‘Fractions of pair types’). In the fields «Fractions of structure types» and «Fractions of pair types» the numbers >70% are colored in orange, the numbers from 40 to 70% are colored in yellow, the numbers from 20% to 40% are colored in green, and the numbers from 10 to 20% are colored in blue.

### Case 2: advanced search with URS

We have compared advanced search options of three different web sites:

1. RCSB PDB (http://www.rcsb.org/pdb/search/adv Search.do?search=new);
2. NDB (http://ndbserver.rutgers.edu/ndbmodule/search/integrated.html);
3. URS (http://server3.lpm.org.ru/urs/struct.py).

Consider for example the following problem: *find all RNA structures containing*

*at least one H-knot (H-type pseudoknot)* ***AND*** *at least one* ***Mg^2+ ^****ion*

***OR***

*at least one H-knot (H-type pseudoknot)* ***AND*** *at least one* ***Ca^2+ ^****ion*

PDB provides opportunity to compose a query only as an AND-junction or as an OR-junction and moreover it has no restrictions on RNA structural features. Despite these shortcomings its capabilities with respect to protein structures are performed at the highest level.

The advanced search at NDB actually has a plenty of parameters related to RNA structures. Moreover at the moment it has more parameters than URS does. Also it allows composing OR-junctions along with AND-junctions. However its AND and OR clauses are placed in predefined order and it is hard to understand their mutual relations. Besides, the advanced search at NDB does not contain any parameters related to pseudoknots.

As for the URS, its advanced search allows one to compose such a query in a couple of clicks. We hope that the search based on disjunctive normal form (i.e. disjunction of conjunctions) is both powerful and understandable.

### Future development

We plan to perform a comparative analysis of programs that annotate base pairs in RNA-containing PDB files. We will consider the four most popular programs, FR3D (35), MC-Annotate (36), RNAView (37) and DSSR (26). According to the analysis the annotation of the base pairs will be refined. In addition, we plan to include in the database annotations of base-phosphate, base-ribose and base stacking contacts and to implement search of such data.

Another direction in the development of the database is related to RNA–protein contacts. We will combine the data on RNA-protein interactions with the annotation of loops and stems and will add data on protein secondary structure.

The list of structural element types available for statistics gathering and further analysis will be significantly enlarged. Along with computation of maximal and minimal value of parameters we will support the construction of histograms, etc. We also plan to implement a multi-step search, allowing user, for example, to form a set of PDB entries, as it is possible now, then form a set of RNA chains in the entries according to additional conditions, and then choose the desired stems from the chains and obtain base pairs statistics on the chosen stems. Such a search will allow the web interface to fully utilize all the features of the database.

We also plan to add some new features to the web interface according to users’ requests. In particular, we plan to add the possibility of using 2D-visualization tools like VARNA (38), PseudoViewer (39) and R-chie (40). We also plan to develop our own visualization tools, e.g. interactive RNA dot-bracket map.

## Conclusion

URSDB is a powerful tool providing researchers with new instruments of RNA analysis compared to the existent tools. The database allows users to select a subset of structures with desired features, and to obtain various statistical data for a selected subset of structures or for all structures. For example, the user can easily get statistics on geometric parameters of base pairs, on structural motifs (base pairs, stems, loops, etc.) or on types of pseudoknots. Users can also view and get information on an individual structure or its selected part, e.g. RNA–protein hydrogen bonds. URSDB employs an original definition of loops that fits both pseudoknot-free and pseudoknotted RNA secondary structures; in case of pseudoknot-free structures the definition coincides with the classical definition. To our knowledge, URSDB is the first database supporting search based on ‘topological’ classification of pseudoknots (28–32) and on extended loop classification.
